# Eight-year trend analysis of malaria prevalence in Kombolcha, South Wollo, north-central Ethiopia: a retrospective study

**DOI:** 10.1186/s13071-018-2654-6

**Published:** 2018-01-24

**Authors:** Daniel Gebretsadik, Daniel Getacher Feleke, Mesfin Fiseha

**Affiliations:** Department of Medical Laboratory Science, College of Medicine and Health Sciences, Wollo University, Dessie, Ethiopia

**Keywords:** Malaria, Prevalence, Ethiopia, Retrospective

## Abstract

**Background:**

Malaria is one of the most serious public health problems in the world, and is a major public health problem in Ethiopia. Over the past years, the disease has been consistently reported as the first leading cause of outpatient visits, hospitalization and death in health facilities across the country. This study aimed to assess malaria prevalence trend in the Kombolcha Health Centre.

**Methods:**

A retrospective study was carried out in the Kombolcha Health Centre, north-central Ethiopia. Malaria cases reported from 2009 to 2016 were carefully reviewed from the laboratory record books. Interventions that had been taken in each year were collected from the district health bureau and head of Kombolcha Health Centre using checklists.

**Results:**

A total of 27,492 blood films were examined from malaria-suspected patients in the Kombolcha Health Centre from 2009 to 2016. Malaria was confirmed and reported in 2066 (7.52%) of the examined blood films with 258 mean annual cases of. Minimum and maximum cases were reported in 2009 and 2010, respectively. *Plasmodium falciparum* and *P. vivax* accounted 60.2% and 35.5% of the cases, respectively. Male patients were more affected (*n* = 1407; 68.1%) than female ones (*n* = 659; 31.89%). The highest malaria prevalence (*n* = 1440; 69.69%) was seen in the 15–45 years age group, followed by those 5–14 years old (*n* = 303; 14.67%), and finally patients under five years old (*n* = 217; 10.5%). Malaria cases were at a peak in spring and reduced in the winter season.

**Conclusion:**

Although the current malaria control strategies are effective in decreasing the morbidity and mortality, malaria is still among major public health problems in Ethiopia. *Plasmodium falciparum* is the dominant species in the study area. However, in recent years *P. vivax* cases are increasing, indicating that attention should also be given to this species*.* The efficacy of chloroquine for *P. vivax* should be evaluated in the study area. Control activities should be continued and scale up.

## Background

Malaria remains one of the most serious public health problems in the world [1, 2]. In 2015, there were an estimated 429,000 malaria-related deaths (range 235,000–639,000) worldwide [3]. Most of these deaths occurred in Africa (92%), Southeast Asia, and the Eastern Mediterranean region [4, 5]. In Ethiopia, malaria is endemic across three-quarters of the landmass, and an estimated 68% of the population lives in these affected areas [4, 6–8]. Over the last decade, the burden of the disease has declined significantly, which may have resulted from the improved coverage of high impact interventions including prompt treatment of cases using artemisinin-based combination therapy (ACT), prevention and control of malaria among pregnant women using intermittent preventive therapy (IPT), and use of vector control methods, i.e. insecticide-treated bed nets (ITNs) and indoor residual spray (IRS) [9–11].

In Ethiopia, *Plasmodium falciparum* and *P. vivax* are the dominant species, with *P. falciparum* accounting for 60% of malaria cases reported in the country [1, 4, 7, 10]. Malaria transmission in the region is seasonal due to climatic and altitude factors [12]. Although some parts of the country have no defined rainfall season, in many areas the number of cases reaches a peak after the main rainfall season, which occurs in July-September each year [6].

In Ethiopia, the prevention and control of malaria involves early diagnosis and prompt treatment, selective vector control using indoor residual spraying (IRS), ITNs and environmental management [4]. Currently, the common drugs used for treatment of *P. falciparum* and *P. vivax* malaria in the study area and throughout the country are artemether/lumefantrine (Coartem) and chloroquine, respectively, since 2004 [4].

According to WHO malaria reports, malaria incidences and deaths decreased by 37% and 60%, respectively, between 2000 and 2015 worldwide [5]. In Ethiopia, the burden of malaria is declining significantly, which could be the result of improved coverage of high impact interventions, such as prompt treatment of cases, prevention and control of malaria among pregnant women using intermittent preventive therapy (IPT), use vector control methods including ITNs, and IRS [10, 11]. However, although there is a significant decline in disease burden, the overall trend of malaria prevalence is not studied or well-documented. Analyzing malaria prevalence trends and the intervention mechanisms employed each year are important for the expansion of intervention strategies or to design new ones to tackle the disease. Here, we analyzed the trends of malaria prevalence and assessed factors affecting malaria in Kombolcha, north-central Ethiopia.

## Methods

### Study area

This study was carried out at Kombolcha Health Centre, Kombolcha town, in the South Wollo zone of the Amhara regional state, north-central Ethiopia. Kombolcha (11°07′N, 39°43′E) is 1875 m above sea level and 376 km northeast of Addis Ababa, which is the capital city of Ethiopia. In 2015, the estimated total population of Kombolcha was 126,144, of whom 65,918 were female. Of the total population, the majority (94,119) of the population lives in urban areas of the town. The mean annual rainfall is 841.1 mm, and the maximum and minimum annual mean temperature of the town is 27.4 °C and 12.9 °C, respectively. The Kombolcha Health Centre was founded in 1989 and is the preferred health institution by the local population and as such has a very high patient load. The district health office and the health centre share the same compound, which helped the health office to supervise and improve the health service. Malaria is the most prevalent seasonal disease in the area, which peaks in October to December.

### Study design

A retrospective malaria data review was conducted to determine the eight-year (2009–2016) malaria prevalence trends from January to December 2016 at the Kombolcha Health Centre.

### Data collection

Eight-year malaria prevalence data were obtained from the Kombolcha Health Centre for the period 2009–2016. Throughout the reviewed period, microscopy was used as the gold standard to confirm *Plasmodium* parasite presence by examination of peripheral smears of stained blood films, as per the WHO protocol. In Ethiopia, all hospitals and health centres follow a standard operating procedure (SOP) for blood smear preparation, staining and blood film examination for malaria parasite detection throughout the country. Kombolcha Health Centre has a very consistent laboratory data recording system. Data were checked for its completeness before analysis; we have found a very small number of records which were incomplete. Also, the laboratory technologists/technicians engaged in malaria microscopy were competent and well-trained in malaria microscopy. Throughout the reviewed period (2009–2016) microscopy was used exclusively for *Plasmodium* spp. detection. In 2012, the treatment guidelines implemented in Ethiopia and the study health centre for *P. falciparum* was changed from chloroquine to artemether/lumefantrine (Coartem).

### Factors affecting malaria trends

Data about factors which can affect malaria trends and about interventions that had been taken in each year were collected from the district health office using checklists. A yearly malaria control and prevention plan and achievements of the district health office were also reviewed. We have also communicated with the head of the health centre and chairperson of the district health office. In past years, malaria control and prevention activities were intensified by all stockholders. The strategies that were applied in the health centre were awareness creation of the community about malaria transmission and control methods, the increment of budget and increased accessibility of ITNs to the community.

### Data analysis

In this study, only consistently recorded demographic information was used in the data analysis. Data were first entered into Excel and then imported into SPSS version 20 (SPSS INC, Chicago, II, USA). Crosstab was used for the frequency distribution of both dependent and independent variables. Data were presented with appropriate figures and tables.

## Results

**Fig. 1:**
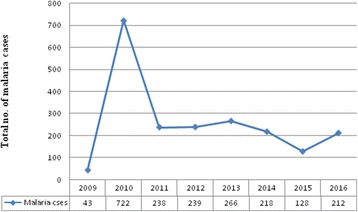
Malaria cases trend in Kombolcha Health Centre from 2009 to 2016

From 2009 to 2016, blood films were prepared and examined from 27,492 malaria-suspected patients at Kombolcha Health Centre. Of these, 2066 (7.52%) were microscopically confirmed malaria cases (annual mean = 258). The minimum (*n* = 43) and maximum (*n* = 722) number of annual malaria cases were reported in 2009 and 2010, respectively (Fig. 1). The highest (*n* = 5568) and lowest (*n* = 2558) number of malaria-suspected patients were examined in 2010 and 2015, respectively. Throughout the reviewed period, malaria was more common in the urban population than the rural population. In both urban and rural people, higher malaria cases were observed in 2010 (Table 1).

**Table 1:** Malaria cases distribution with regard to residences in Kombolcha Health Centre from 2009 to 2016

Year	Total no. of blood films examined	Total no. of cases (%)	Residence type	
			Urban	Rural
2009	2999	43 (1.43)	30	13
2010	5568	722 (12.97)	494	228
2011	3875	238 (6.14)	170	68
2012	3383	239 (7.06)	146	93
2013	3031	266 (8.78)	146	120
2014	3115	218 (7.0)	138	80
2015	2558	128 (5.0)	91	37
2016	2963	212 (7.15)	148	64

*Plasmodium falciparum* was the most frequently reported species and accounted for 1243 (60.2%) cases while *P. vivax* accounted for 734 (35.5%) cases. Mixed *P. falciparum*/*P. vivax* infections accounted for 4.3% of confirmed cases (*n* = 89). *Plasmodium falciparum* was also the most commonly reported species in all patient age groups, with the maximum number of 868 (42%) cases reported in the 15–45 years age group. *Plasmodium falciparum* and *P. vivax* were most frequently recorded from patients 15–45 years of age (Table 2).

**Table 2:** The prevalence of *Plasmodium* spp. in different age groups in Kombolcha Health Centre from 2009 to 2016

Age category	*P. falciparum n* (%)	*P. vivax n* (%)	Mixed infection (*Pf* + *Pv*) *n* (%)
< 5	125 (10.1)	82 (11.2)	9 (10.1)
5–14	186 (15.0)	100 (13.6)	17 (19.1)
15–45	868 (69.8)	513 (69.9)	59 (66.3)
> 45	64 (5.1)	39 (5.3)	4 (4.5)

*Abbreviations: Pv Plasmodium vivax*, *Pf Plasmodium falciparum*

**Fig. 2:**
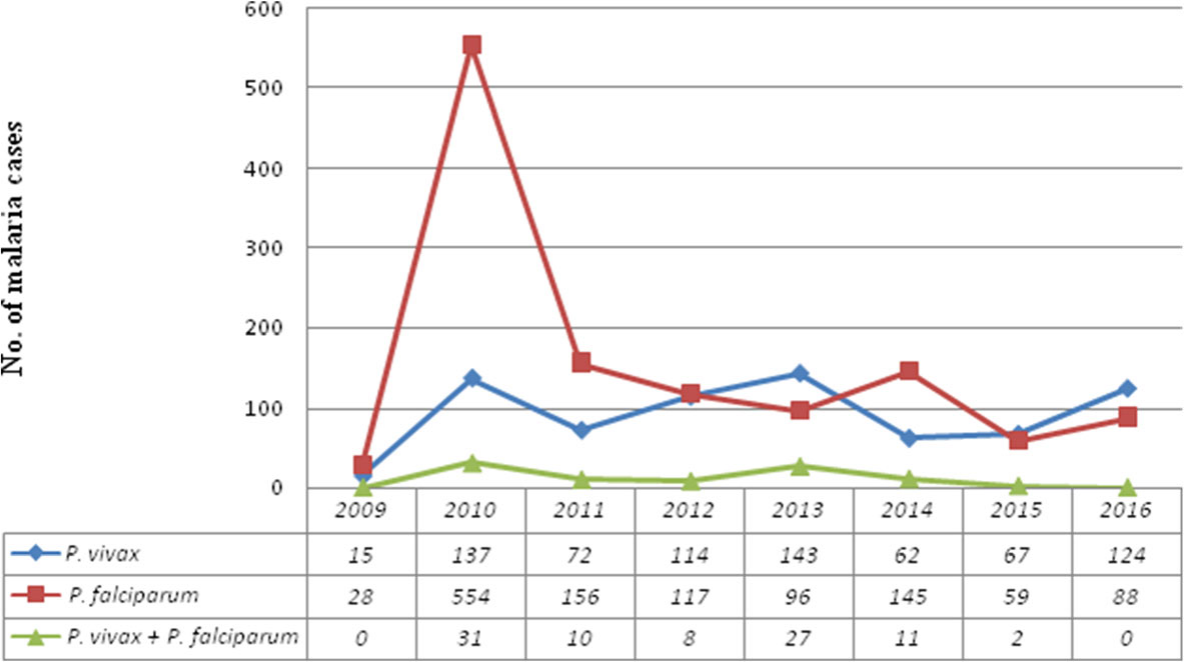
*Plasmodium* species trend in Kombolcha Health Centre from 2009 to 2016

*Plasmodium falciparum*, *P. vivax* and mixed infections showed an increase in 2010 compared to cases reported in 2009. *Plasmodium vivax* cases increased by 39 cases from 2012 to 2013, and by 50% from 2015 to 2016, while *P. falciparum* cases were reduced by 21 cases from 2012 to 2013 (Fig. 2).

**Fig. 3:**
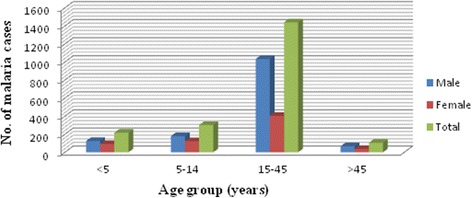
The distribution of malaria cases in different age groups and sex in Kombolcha Health Centre from 2009 to 2016

Out of 2066 microscopically confirmed malaria cases, 1407 (68.1%) were reported in males and 659 (31.89%) in females with a male to female ratio of 2.14. In the reviewed period, all age groups were infected by malaria with the higher prevalence (1440; 69.69%) occurring among the 15–45 years age group, followed by 5–14 years age group (303; 14.67%) and the under 5 years of age group (217; 10.5%). The lowest prevalence of infection was reported in patients older than 45 years of age (107; 5.18%). Males were more affected than females in all age groups (Fig. 3).

### Seasonal variation of malaria in Kombolcha Health Centre

**Fig. 4:**
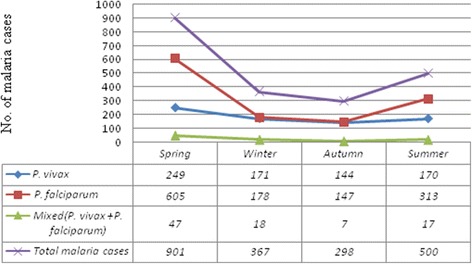
The distribution of *Plasmodium* species in different seasons in Kombolcha Health Centre from 2009 to 2016

The prevalence of malaria for the four seasons in Ethiopia was analyzed. Except the year 2009 where malaria cases were significantly low, malaria cases occurred in all months and seasons. In 2009, malaria cases were not observed in June, and the only single case was recorded in September, October, March, April and May. The maximum number of malaria cases was reported in spring (September-November) and the minimum was during winter (December–February). *Plasmodium vivax*, *P. falciparum* and mixed infections were high in the spring and lower in winter (Fig. 4).

## Discussion

The present study demonstrated that malaria prevalence fluctuated annually, with the minimum and maximum number of cases recorded in 2009 and 2010, respectively. The reduction of malaria cases in 2009 may be attributed to the combined effort of the society and other stakeholders. In 2009, the district health office yearly plan to distribute ITNs to all people who were at risk was successful. There was also a campaign to create awareness and environmental protection. Also, due to the devastating problem of malaria before 2009, all stakeholders exerted maximal effort to control the disease. Government and non-government organizations participated in human capacity development. The maximum number of malaria cases recorded in 2010 may be due to community misconception surrounding malaria prevention and control. Although successful in controlling the disease in 2009, it is conceivable, due to poor community education, that local populations perceived this affect would be consistently maintained without constant implementation programs/efforts. This highlights the need for continuous and collaborative efforts to control, prevent and (potentially) eradicate malaria. Although the number of malaria cases was at its highest in 2010 and has fluctuated annually until 2016, in recent years it has decreased in the study area. This may be due to the effectiveness of national strategies for malaria control and prevention. Here, malaria prevalence is lower than the retrospective studies conducted in Kola Diba and Arsi Negelle Health Centres [4, 13]. This might be due to climatic and altitude difference, and laboratory personnel performance difference in malaria parasite detection. The increase in malaria cases might also be due to the occurrence of malaria epidemics. This was in agreement with the study conducted in Kola Diba Health Centre [4]. However, this report was contrasted with a report from Arsi Negelle [13].

The Ethiopian Ministry of Health gave great attention to reduce malaria cases to zero and avoid malaria-related health problems; this resulted in a reduction of malaria cases in the study area. Malaria control and prevention strategies are being implemented in full scale in all parts of the country. According to the plan and achievement of the district health office of the study area, in recent years there there has been an increased use and accessibility to ITNs, awareness creation, budget increment and training of laboratory personnel to enhance their performance of malaria parasites detection.

In the present study, the number of malaria cases in 2011 and 2012 were nearly almost the same; in 2013, the number of cases increased from 238 to 266. The minimum number of cases was observed in 2015; that might be due to shortage of rain before the start of major and minor malaria transmission period of the country.

*Plasmodium falciparum* and *P. vivax* were the dominant species in the study area. This was in agreement with the national profile of *Plasmodium* species [1, 14]. In our study, the prevalence of *Plasmodium* species varied from year to year. In recent years, especially in 2015 and 2016, there was a decrease in *P. falciparum* cases and an increase in *P. vivax* cases. This might indicate a trend shift of *Plasmodium* species in the study area. This was in agreement with a study conducted in Arsi Negelle where *P. vivax* was dominated by *P. falciparum* [13]. Stakeholders have given much attention to tackle *P. falciparum,* due to its clinical complications and drug-resistant threat. In contrast, less attention has been given to other *Plasmodium* species. As a result, *P. vivax* drug resistance to chloroquine might be developing in the study area; however, there is no study conducted on the drug efficacy of chloroquine.

In the study area, the number of malaria cases peaked in spring, followed by summer. These two seasons are the major and minor transmission periods in Ethiopia, respectively. The major transmission period is due to the heavy rains from June to September which create suitable environment for the breading of *Anopheles* mosquitoes. The minor transmission period is as a result of small shower rains from February to March. Although malaria transmission is usually associated with rainy seasons, in the present study malaria cases were also significant in winter and autumn, indicating that climatic and environmental factors other than rainfall can also determine the occurrence of malaria.

In this study, the prevalence of malaria was higher in males (68.1%) than in females (31.89%). This might be due to the lifestyle and occupation of males. Males are usually involved in industrial, agricultural and day labour. In these work environments, there might be suitable environments that favour mosquito breeding. The finding is in agreement with a study conducted in India [15, 16]. Regarding age groups, the 15–45 years age group were highly affected (69.69%) followed by the 5–14 years age group (14.67%). This was in agreement with a study conducted in Kola Diba Health Centre [4]. However, in contrast to this finding, the study conducted in Wolita showed high malaria positivity in 5–14 year olds [17]. The reason why malaria affects the 15–45 age group in Kombolcha might be because these age groups are productive and as a result they are actively involved in industrial and agricultural activities. This can increase the exposure of these groups of the population to *Anopheles* mosquito bites, which can transmit *Plasmodium* parasites.

In summary, environmental, biological, socio-economical and policy changes may have attributed to the fluctuating trend of malaria prevalence observed in the study area, such as the immunity of the population, environmental and climatic changes, infrastructures in the study area, societal awareness, accessibility of malaria control and prevention tools and the socio-economic changes of the society.

The strength of this study lies in the fact that malaria data were collected by trained laboratory technologists. The study has also investigated the factors associated with the trends of malaria prevalence by collecting comprehensive information from responsible bodies. The limitation of the study was incompleteness of recorded data in the laboratory registration book of the health centre. Due to this reason, we excluded five registered data.

## Conclusions

Although the current malaria control strategies are effective in decreasing patient morbidity and mortality, malaria is still among the major public health problems in Ethiopia. *Plasmodium falciparum* is the dominant species in the study area. However, in recent years *P. vivax* cases are increasing. This indicates that attention should also be given to *P. vivax* especially with regard to drug resistance and other aspects of malaria control strategies. Control activities should be continued and scale up in the study area.

## Abbreviations

IRS: indoor residual spraying
ITNs: insecticide-treated nets
SOP: standard operating procedure
WHO: World Health Organization
